# Impact of Body Weight Dynamics Following Intentional Weight Loss on Fracture Risk: Results from The Action for Health in Diabetes Study

**DOI:** 10.1002/jbm4.10086

**Published:** 2018-10-25

**Authors:** Kristen M Beavers, Rebecca H Neiberg, Karen C Johnson, C Hunter Davis, Ramon Casanova, Ann V Schwartz, Carolyn J Crandall, Cora E Lewis, Xavier Pi‐Sunyer, Stephen B Kritchevsky, the Look AHEAD Research Group

**Affiliations:** ^1^ Department of Health and Exercise Science Wake Forest University Winston‐Salem NC USA; ^2^ Department of Biostatistics and Data Science Wake Forest School of Medicine Winston‐Salem NC USA; ^3^ Department of Preventive Medicine University of Tennessee Health Science Center Memphis TN USA; ^4^ Department of Epidemiology and Biostatistics University of California San Francisco San Francisco CA USA; ^5^ Department of Internal Medicine University of California Los Angeles Los Angeles CA USA; ^6^ Department of Medicine, Division of Preventive Medicine University of Alabama at Birmingham Birmingham AL USA; ^7^ Department of Medicine Columbia University New York NY USA

**Keywords:** WEIGHT CHANGE, WEIGHT VARIABILITY, FRACTURE, BMD, TYPE 2 DIABETES

## Abstract

The purpose of this study is to explore the impact of body weight change following intentional weight loss on incident fracture and bone mineral density (BMD) in overweight and obese adults with diabetes. A total of 1885 individuals with type 2 diabetes (baseline age: 58.5 ± 6.7 years, 58% women, body mass index: 35.7 ± 6.0 kg/m^2^) who participated in the Look AHEAD study and lost any weight 1 year after being randomized to an intensive lifestyle intervention were assessed. Body weight was measured annually and participants were categorized as weight regainers, weight cyclers, or continued losers/maintainers based on a ±3% annual change in weight from year 1 to year 4. Adjudicated overall fracture incidence was captured from years 4 through 13 (median follow‐up duration 11.5 years). Hip and spine BMD was assessed in a subset of participants at baseline, year 4 (*n* = 468), and year 8 (*n* = 354), using dual‐energy X‐ray absorptiometry. Cox proportional hazards and linear regression models, adjusted for relevant covariates, were performed for fracture and BMD outcomes, respectively. Fifty‐eight percent, 22%, and 20% of participants were classified as weight regainers, weight cyclers, and continued losers/maintainers, respectively; and 217 fractures (men *n* = 63; women *n* = 154) were recorded during the follow‐up period. There were no statistically significant differences in total incident fracture rates for weight regainers (HR [95% CI]: 1.01 [95% CI, 0.71 to 1.44]) or weight cyclers (HR [95% CI]: 1.02 [95% CI, 0.68 to 1.53]) when compared to continued losers/maintainers (*p* = 0.99). Similarly, follow‐up BMD estimates did not significantly vary by weight pattern group, although consistent trends for lowered BMD in the hip region were noted for continued losers/maintainers and weight cyclers compared with weight regainers. Patterns of weight change in the 3 years following 1 year of intentional weight loss were not associated with subsequent fracture or significantly reduced BMD in this cohort of overweight and obese adults with type 2 diabetes. © 2018 The Authors. *JBMR Plus* Published by Wiley Periodicals, Inc. on behalf of American Society for Bone and Mineral Research.

## Introduction

Osteoporotic fracture is a serious and costly clinical problem associated with type 2 diabetes.^1^ Overweight or obese individuals with diabetes are often prescribed weight loss to improve glycemic control,^2^ yet weight loss may further augment fracture risk.^3^ Indeed, recent results from the Look AHEAD study suggest 6% to 9% weight loss achieved and maintained over nearly a decade is associated with significantly reduced bone mineral density (BMD)^4^, ^5^ and increased risk of hip, pelvis, and upper arm fracture.^6^ Data also suggest that bone mass is not recovered when lost weight is regained,^7^, ^8^ raising concern about the long‐term impact of repeated weight loss attempts on bone health. Given the well‐known recidivism of obesity,^9^ surprisingly little is known about the effect of weight regain or weight cycling on incident fracture risk. Limited observational data do link weight variability^10^, ^11^ and self‐reported weight cycling^12^, ^13^ with higher fracture incidence; however, the long‐term impact of objectively measured patterns of weight change on fracture risk following a structured weight loss program has not been assessed. Data collected in the Look AHEAD (Action for Health in Diabetes; Clinicaltrials.gov Identifier: NCT00017953) study provide a unique opportunity to address this question. Using measured weights over a 4‐year period, we aimed to explore associations of three patterns of weight change following intentional weight loss (ie, weight regainers, weight cyclers, and continued losers/maintainers) on fracture risk and change in BMD occurring over the next decade.

## Materials and Methods

The Look AHEAD Study was a multicenter, randomized controlled trial designed to determine whether intentional weight loss reduces cardiovascular morbidity and mortality in overweight individuals with type 2 diabetes. The study was approved by local Institutional Review Boards and all participants provided informed consent. Details on the study design^14^ and baseline characteristics^15^ have previously been published, along with treatment effects on BMD^4^, ^5^ and fracture.^6^ The primary analysis of this work includes 1885 (of 2570) Intensive Lifestyle Intervention (ILI) participants who were successful at losing any weight during the first year of the study (*n* = 2290) and had at least two follow‐up weights (excluded *n* = 301), no fractures prior to year 4 (excluded *n* = 77), and non‐missing covariate data (excluded *n* = 27).

### Exposure assessment: weight patterns

Weight was measured annually by certified clinic staff, masked to intervention assignment, using a Tanita BWB 800 digital scale (Tanita, Willobrook, IL). Based on the first 3 years of follow‐up (years 1 through 4), participants were classified into three categories: (i) weight regainers, (ii) weight cyclers, and (iii) continued losers/maintainers, using patterns previously employed in the Look AHEAD study^16^ and a clinically meaningful^17^ ±3% weight change threshold.

### Primary outcome assessment: incident fracture

Centrally adjudicated incident fractures included in this analysis occurred between year 4 and the end of the Look AHEAD‐Continuation phase, with maximum follow‐up time of 13.2 years (median, 11.5 years). As described,^6^ overall fracture included adjudicated hand (not finger), lower arm or wrist, elbow, upper arm, shoulder, clavicle, spine or back, tailbone, pelvis, hip, upper leg, knee, lower leg or ankle, and foot (not toe); we also examined a composite variable for the first occurrence of hip, pelvis, or upper arm fracture (not including clavicle or scapula).

### Secondary outcome assessment: change in regional BMD

Total hip, femoral neck, and lumbar spine BMD were assessed at five of the 16 Look AHEAD clinical sites using Hologic fan beam densitometers (DXA), as described.^4^, ^5^ The BMD study sample was derived using the same inclusion criteria described for the incident fracture sample, and limited to participants with baseline and year 4 (*n* = 468) or year 8 (*n* = 354) regional DXA scans.

### Covariates

Self‐reported characteristics (ie, age, gender, race/ethnicity, smoking status, and alcohol consumption) and medical history were assessed using standardized questionnaires. Height was measured in duplicate using a stadiometer and body mass index (BMI) was calculated as weight in kilograms divided by height in meters squared. Participants brought current prescription medications to update medication records, with bone negative medications defined as: loop diuretics, selective serotonin reuptake inhibitors (SSRIs), thyroid hormones, oral steroids such as prednisone, tricyclic antidepressants, and thiazolidinedione (TZDs); and bone positive medications defined as: androgens (anabolic steroids), calcium, antacids containing calcium, and antiresorptive agents such as bisphosphonates, calcitonin nasal spray, estrogens, and selective estrogen receptor modulators (SERMs). Fasting blood specimens were analyzed by the Central Biochemistry Laboratory (Northwest Lipid Research Laboratories, University of Washington, Seattle, WA, USA) using standardized laboratory procedures for measuring glycated hemoglobin (HbA1c). Depressive symptoms were assessed using the Beck Depression Inventory (BDI; score range 0 to 63), with higher scores indicating more symptoms of depression.

### Statistical methods

Descriptive statistics were calculated overall and by weight pattern classification at baseline. Cox proportional hazards models, both unadjusted and adjusted for relevant baseline covariates (including: age category, gender, race, BMI category, bone‐positive medication use, and bone‐negative medication use, history of arthritis, HbA1c, smoking status, alcohol consumption, diabetes duration, and BDI score) were performed for incident overall and hip, pelvis, or upper arm fracture outcomes. Because fracture risk is elevated in older women, a formal test of interaction between age, gender, and weight pattern category was conducted to inform whether stratified analyses should be performed. Differences in hip, femoral neck, and lumbar spine BMD at years 4 and 8 were assessed in a subset of participants using linear regression models, adjusting for the same covariates described above plus baseline value of the outcome, and presented as least square means and standard errors. All statistical analyses were executed using SAS version 9.4 (SAS Institute, Inc., Cary, NC, USA).

## Results

### Participant characteristics

Baseline descriptive characteristics, detailed by group and overall, are presented in Table 1. As shown, average age was 58.5 ± 6.7 years, 58% were female, 64% were white, and BMI was 35.7 ± 6.0 kg/m^2^. Fifty‐eight percent, 22% and 20% of participants were classified as weight regainers, weight cyclers, and continued losers/maintainers, respectively. On average, by the year 4 visit, regainers gained 8.3 ± 5.5 kg, weight cyclers gained 1.1 ± 7.0 kg, and continued losers/maintainers lost 2.9 ± 6.0 kg from the year 1 weight. Weight cyclers were more likely to be between the ages of 50 and 59 years, female, Hispanic, and using bone‐positive medications, than other categories (all *p* ≤ 0.02). Participants in the DXA analyses were more likely to be female (*p* < 0.01), of ethnic minority (*p* < 0.01), and slightly younger (*p* = 0.05) when compared to the main analysis sample; however, the distribution of weight pattern categories within the DXA subset was similar to the larger study sample (data not shown).

**Table 1:** Baseline Characteristics Overall and by Weight Pattern Category Among Look AHEAD Participants Randomized to Intensive Lifestyle Intervention Who Lost Weight in the First Year

Characteristic	Overall	Weight regainer	Weight cycler	Continued loser or maintainer	*p*
Total, *n* (%)	1885 (100)	1086 (58)	418 (22)	381 (20)	
Age category (years), *n* (%)					0.02
<50 years	205 (11)	117 (11)	52 (12)	36 (9)	
50–59 years	890 (47)	495 (46)	223 (53)	172 (45)	
60–69 years	669 (35)	407 (37)	121 (29)	141 (37)	
70+ years	121 (6)	67 (6)	22 (5)	32 (8)	
Female gender, *n* (%)	1090 (58)	597 (55)	286 (68)	207 (54)	<0.01
Race/ethnicity, *n* (%)					<0.01
African American	305 (16)	162 (15)	65 (16)	78 (20)	
Hispanic	229 (12)	119 (11)	61 (15)	49 (13)	
White	1214 (64)	745 (69)	259 (62)	210 (55)	
Other	137 (7)	60 (6)	33 (8)	44 (12)	
BMI category (kg/m^2^), *n* (%)					0.05
25–27	59 (3)	30 (3)	12 (3)	17 (4)	
27–30	251 (13)	149 (14)	43 (10)	59 (15)	
30–35	675 (36)	412 (38)	143 (34)	120 (32)	
35–40	486 (26)	266 (24)	113 (27)	107 (28)	
40+	414 (22)	229 (21)	107 (26)	78 (20)	
Bone‐negative medication use, *n* (%)^a^	851 (45)	493 (45)	192 (46)	166 (44)	0.77
Bone‐positive medication use, *n* (%)^b^	471 (25)	261 (24)	125 (30)	85 (22)	0.03
Presence of arthritis, *n* (%)	771 (41)	426 (39)	174 (42)	171 (45)	0.15
HbA1c (%), mean ± SD	7.2 ± 1.1	7.1 ± 1.1	7.3 ± 1.1	7.3 ± 1.1	<0.01
Smoking status, *n* (%)					0.17
Never	950 (50)	533 (49)	233 (56)	184 (48)	
Past	852 (45)	506 (46)	168 (40)	178 (47)	
Present	83 (5)	47 (4)	17 (4)	19 (5)	
Alcohol consumption, *n* (%)					0.08
None/week	1279 (68)	720 (66)	295 (71)	264 (69)	
1–3/week	358 (19)	219 (20)	80 (19)	59 (15)	
4+/week	248 (13)	147 (14)	43 (10)	58 (15)	
Diabetes duration (years), mean ± SD	6.6 ± 6.6	6.6 ± 6.6	6.4 ± 6.7	7.0 ± 6.4	0.36
Beck Depression Inventory score (0–63), mean ± SD	5.3 ± 5.0	5.1 ± 4.9	5.7 ± 5.2	5.2 ± 4.8	0.14

Weight change pattern during the first 4 years of follow‐up.

SSRI = selective serotonin reuptake inhibitor; TZD = thiazolidinedione; SERM = selective estrogen receptor modulator.

^a^ Bone‐negative medications are defined as: loop diuretics, SSRIs, thyroid hormones, oral steroids such as prednisone, tricyclic antidepressants, and TZDs.

^b^ Bone‐positive medications are defined as: androgens (anabolic steroids), calcium, antacids containing calcium, and antiresorptive agents such as bisphosphonates, calcitonin nasal spray, estrogens, and SERMs.

### Association between weight patterns and incident fracture

**Figure 1:**
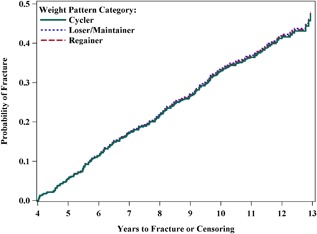
Cumulative hazard curve using predicted values from Cox proportional hazard regression fully‐adjusted model displaying weight pattern category by probability of fracture and years to fracture.

Over a total of 11.5 years of follow‐up, 217 fractures (men *n* = 63; women *n* = 154) were recorded overall. Figure 1 presents Kaplan‐Meier curves of incident overall fracture by weight pattern category. There were no statistically significant differences in total incident fracture rates for weight regainers (HR [95% CI]: 1.01 [95% CI, 0.71 to 1.44]) or weight cyclers (1.02 [95% CI, 0.68 to 1.53]) when compared to continued losers/maintainers (*p* = 0.99). Similar results were observed for incident hip, pelvis, or upper arm fracture (data not shown). The interaction between age, gender, and weight pattern category was not significant (*p* = 0.75), thus stratified analyses were not pursued.

### Association between weight patterns and regional BMD

As with incident fracture, no significant associations were observed between weight pattern classification and year 4 and year 8 regional BMD at any site. Trends for lower BMD at the femoral neck and total hip were observed in continued losers/maintainers and weight cyclers compared with weight regainers, yet they did not attain statistical significance. Specifically, by year 8, femoral neck BMD was reduced from baseline by −0.050 ± 0.012 g/cm^2^ (−4.5%), −0.055 ± 0.012 g/cm^2^ (−3.9%), and −0.039 ± 0.010 g/cm^2^ (−3.0%) in continued losers/maintainers, weight cyclers, and weight regainers, respectively (*p* = 0.11). Likewise, by year 8, total hip BMD was reduced by −0.046 ± 0.012 g/cm^2^ (−3.9%), −0.042 ± 0.012 g/cm^2^ (−3.3%), and −0.032 ± 0.011 g/cm^2^ (−1.9%) from baseline values in continued losers/maintainers, weight cyclers, and weight regainers, respectively (*p* = 0.21). No signal was observed for change in lumbar spine BMD (continued losers/maintainers: +0.033 ± 0.015 g/cm^2^, weight cyclers: +0.018 ± 0.015 g/cm^2^, and weight regainer: +0.023 ± 0.013 g/cm^2^; *p* = 0.48).

## Discussion

There is increasing interest in understanding the skeletal effects of obesity and diabetes, particularly in the context of weight loss. As reported, randomization to an intensive lifestyle intervention, resulting in weight loss for most participants, was associated with modest bone loss^4^, ^5^ and increased risk of fractures occurring at the hip, pelvis, and upper arm.^6^ Here we report that, among those randomized to the lifestyle intervention who lost weight during the first year, patterns of weight change in the subsequent 3 years were not associated with incident fracture or significantly reduced BMD; however, data suggest a trend toward lowered BMD at the hip among continued losers/maintainers and weight cyclers, compared to weight regainers.

We are aware of only four studies examining the effect of weight variability^10^, ^11^ or cycling^12^, ^13^ on fracture risk to date, all of which report positive associations. The earliest reports, examining weight variability (defined using the root mean square error) and incident hip fracture, suggest a 50% to 270% increased risk for those in the highest quartile of weight variability, compared to the lowest. More recent studies led by Søgaard and colleagues^12^, ^13^ extend these findings to weight cycling, demonstrating increased risk of forearm fracture in men^12^ and nonvertebral fracture in women^13^ who self‐reported multiple weight loss episodes (ie, ≥4 in men and ≥11 in women). In contrast, relative to continued weight loss or weight loss maintenance, we did not observe an association between a single bout of weight regain or weight cycling on fracture incidence, thereby adding equipoise to a limited evidence base. Certainly, discrepancies may be due to differing definitions used for weight cycling, as well as the lower number of absolute weight cycles possible in our study.

To our knowledge, our study is the first to assess the effect of weight patterns dynamics following *intentional* weight loss on incident fracture; although the effect of weight change following voluntary weight loss on BMD has been assessed in a handful of studies. Some, but not all,^18^, ^19^ studies suggest that the well‐described 2% decline in BMD with 10% weight loss continues progressively despite weight loss maintenance,^20^ and does not return with weight regain.^7^, ^8^ Trends observed in our BMD data are in general agreement with prior reports, although results were nonsignificant. Importantly, if true, these observations suggest that skeletal remodeling is affected by factors other than change in loading forces, and may imply that transient weight loss attempts have lasting catabolic effects on the skeleton.

Strengths of this study include use of objectively measured weights to assess changing weight dynamics following intentional weight loss, as well as adjustment for multiple, relevant covariates. Despite these design strengths, Look AHEAD was not designed to detect differences in incident fracture rates between weight pattern classifications; thus, our analyses may have been underpowered. Certainly, DXA data, which were only available in a subset, are meant to be hypothesis generating, rather than confirmatory. A general limitation in this field of research is the lack of an operational definition for weight cycling. Although we used patterns previously employed in the Look AHEAD study^16^ and a clinically meaningful^17^ ±3% weight change threshold, conclusions may differ by weight pattern category definition used. Last, extrapolation of results should be limited to similar populations, particularly with regard to age and disease status.

In conclusion, in this cohort of overweight and obese adults with type 2 diabetes experiencing intentional weight loss over a year, we did not observe a significant association between subsequent patterns of weight change and incident fracture. Overall, findings temper concerns regarding weight cycling following intentional weight loss on fracture risk, at least in the short term, although certainly more work in this area is needed.

## Disclosures

All authors state that they have no conflicts of interest.

## Look AHEAD Research Group at End of Continuation

### Clinical sites

The Johns Hopkins University: Frederick L. Brancati, MD, MHS^1*^; Jeanne M. Clark, MD, MPH^1^ (Co‐Principal Investigators); Lee Swartz^2^; Jeanne Charleston, RN^3^; Lawrence Cheskin, MD^3^; Richard Rubin, PhD^3*^; Jean Arceci, RN; David Bolen; Danielle Diggins; Mia Johnson; Joyce Lambert; Sarah Longenecker; Kathy Michalski, RD; Dawn Jiggetts; Chanchai Sapun; Maria Sowers; Kathy Tyler.

*deceased

Pennington Biomedical Research Center: George A. Bray, MD^1^; Allison Strate, RN^2^; Frank L. Greenway, MD^3^; Donna H. Ryan, MD^3^; Donald Williamson, PhD^3^; Timothy Church, MD^3^; Catherine Champagne, PhD, RD; Valerie Myers, PhD; Jennifer Arceneaux, RN; Kristi Rau; Michelle Begnaud, LDN, RD, CDE; Barbara Cerniauskas, LDN, RD, CDE; Crystal Duncan, LPN; Helen Guay, LDN, LPC, RD; Carolyn Johnson, LPN, Lisa Jones; Kim Landry; Missy Lingle; Jennifer Perault; Cindy Puckett; Marisa Smith; Lauren Cox; Monica Lockett, LPN.

The University of Alabama at Birmingham: Cora E. Lewis, MD, MSPH^1^; Sheikilya Thomas, PhD,MPH^2^; Monika Safford, MD^3^; Stephen Glasser, MD^3^; Vicki DiLillo, PhD^3^; Gareth Dutton, PhD, Charlotte Bragg, MS, RD, LD; Amy Dobelstein; Sara Hannum; Anne Hubbell, MS; Jane King, MLT; DeLavallade Lee; Andre Morgan; L. Christie Oden; Janet Wallace, MS; Cathy Roche, PhD, RN, BSN; Jackie Roche; Janet Turman.

Harvard Center

*Massachusetts General Hospital*: David M. Nathan, MD^1^; Enrico Cagliero, MD^3^; Heather Turgeon, RN, BS, CDE^2^; Barbara Steiner, EdM; Valerie Goldman, MS, RDN^2^; Linda Delahanty, MS, RDN^3^; Ellen Anderson, MS, RDN^3^; Laurie Bissett, MS, RDN; Christine Stevens, RN; Mary Larkin, RN; Kristen Dalton, BS, Roshni Singh, BS.

*Joslin Diabetes Center*: Edward S. Horton, MD^1^; Sharon D. Jackson, MS, RD, CDE^2^; Osama Hamdy, MD, PhD^3^; A. Enrique Caballero, MD^3^; Sarah Bain, BS; Elizabeth McKinney, BSN, RN; Barbara Fargnoli, MS,RD; Jeanne Spellman, BS, RD; Kari Galuski, RN; Ann Goebel‐Fabbri, PhD; Lori Lambert, MS, RD; Sarah Ledbury, MEd, RD; Maureen Malloy, BS; Kerry Ovalle, MS, RCEP, CDE.

*Beth Israel Deaconess Medical Center*: George Blackburn, MD, PhD^1*^ Christos Mantzoros, MD, DSc^3^; Ann McNamara, RN.

*deceased

University of Colorado Anschutz Medical Campus: James O. Hill, PhD^1^; Marsha Miller, MS RD^2^; Holly Wyatt, MD^3^, Brent Van Dorsten, PhD^3^; Judith Regensteiner, PhD^3^; Debbie Bochert; Gina Claxton‐Malloy RD Ligia Coelho, BS; Paulette Cohrs, RN, BSN; Susan Green; April Hamilton, BS, CCRC; Jere Hamilton, BA; Eugene Leshchinskiy; Loretta Rome, TRS; Terra Thompson, BA, Kirstie Craul, RD, CDE; Cecilia Wang, MD.

Baylor College of Medicine: John P. Foreyt, PhD^1^; Rebecca S. Reeves, DrPH, RD^2^; Molly Gee, MEd, RD^2^; Henry Pownall, PhD^3^; Ashok Balasubramanyam, MBBS^3^; Chu‐Huang Chen, MD, PhD^3^; Peter Jones, MD^3^; Michele Burrington, RD, RN; Allyson Clark Gardner, MS, RD; Sharon Griggs; Michelle Hamilton; Veronica Holley; Sarah Lee; Sarah Lane Liscum, RN, MPH; Susan Cantu‐Lumbreras; Julieta Palencia, RN; Jennifer Schmidt; Jayne Thomas, RD; Carolyn White; Charlyne Wright, RN; Monica Alvarez, PCT.

The University of Tennessee Health Science Center

*University of Tennessee East*: Karen C. Johnson, MD, MPH^1^; Karen L. Wilson, BSN^2^; Mace Coday, PhD^3^; Beate Griffin, RN, BS; Donna Valenski; Polly Edwards; Brenda Fonda; Kim Ward.

*University of Tennessee Downtown*: Helmut Steinburg, MD^3^; Carolyn Gresham, BSN^2^; Moana Mosby, RN; Debra Clark, LPN; Donna Green RN; Abbas E. Kitabchi, PhD, MD (retired).

University of Minnesota: Robert W. Jeffery, PhD^1^; Tricia Skarphol, MA^2^; John P. Bantle, MD^3^; J. Bruce Redmon, MD^3^; Richard S. Crow, MD^3^; Scott J. Crow, MD^3^; Manami Bhattacharya, BS; Cindy Bjerk, MS, RD; Kerrin Brelje, MPH, RD; Carolyne Campbell; Mary Ann Forseth, BA; Melanie Jaeb, MPH, RD; Philip Lacher, BBA; Patti Laqua, BS, RD; Birgitta I. Rice, MS, RPh, CHES; Ann D. Tucker, BA; Mary Susan Voeller, BA.

St. Luke's Roosevelt Hospital Center: Xavier Pi‐Sunyer, MD^1^; Jennifer Patricio, MS^2^; Carmen Pal, MD^3^; Lynn Allen, MD; Janet Crane, MA, RD, CDN; Lolline Chong, BS, RD; Diane Hirsch, RNC, MS, CDE; Mary Anne Holowaty, MS, CN; Michelle Horowitz, MS, RD; Les James; Raashi Mamtani, MS.

University of Pennsylvania: Thomas A. Wadden, PhD^1^; Barbara J. Maschak‐Carey, MSN, CDE^2^; Robert I. Berkowitz, MD^3^; Gary Foster, PhD^3^; Henry Glick, PhD^3^; Shiriki Kumanyika, PhD RD, MPH^3^; Yuliis Bell, BA; Raymond Carvajal, PsyD; Helen Chomentowski; Renee Davenport; Lucy Faulconbridge, PhD; Louise Hesson, MSN, CRNP; Sharon Leonard, RD; Monica Mullen, RD, MPH.

University of Pittsburgh: John M. Jakicic, PhD^1^; David E. Kelley, MD^1^; Jacqueline Wesche‐Thobaben, RN, BSN, CDE^2^; Daniel Edmundowicz, MD^3^; Lin Ewing, PhD, RN^3^; Andrea Hergenroeder, PhD, PT, CCS^3^; Mary L. Klem, PhD, MLIS^3^; Mary Korytkowski, MD^3^; Andrea Kriska, PhD^3^; Lewis H. Kuller, MD, DrPH^3^; Amy D. Rickman, PhD, RD, LDN^3^; Rose Salata, MD^3^; Monica E. Yamamoto, DrPH, RD, FADA^3^; Janet Bonk, RN, MPH; Susan Copelli, BS, CTR; Rebecca Danchenko, BS; Tammy DeBruce, BA; Barbara Elnyczky; David O. Garcia, PhD; George A. Grove, MS; Patricia H. Harper, MS, RD, LDN; Susan Harrier, BS; Diane Heidingsfelder, MS, RD, CDE, LDN; Nicole L. Helbling, MS, RN; Diane Ives, MPH; Janet Krulia, RN, BSN, CDE; Juliet Mancino, MS, RD, CDE, LDN; Anne Mathews, PhD, RD, LDN; Lisa Martich, BS, RD, LDN; Meghan McGuire, MS; Tracey Y. Murray, BS; Anna Peluso, MS; Karen Quirin; Jennifer Rush, MPH; Joan R. Ritchea; Linda Semler, MS, RD, LDN; Karen Vujevich, RN‐BC, MSN, CRNP; Kathy Williams, RN, MHA; Donna L. Wolf, PhD.

The Miriam Hospital/Brown Medical School: Rena R. Wing, PhD^1^; Renee Bright, MS^2^; Vincent Pera, MD^3^; Deborah Tate, PhD^3^; Amy Gorin, PhD^3^; Kara Gallagher, PhD^3^; Amy Bach, PhD; Barbara Bancroft, RN, MS; Anna Bertorelli, MBA, RD; Richard Carey, BS; Tatum Charron, BS; Heather Chenot, MS; Kimberley Chula‐Maguire, MS; Pamela Coward, MS, RD; Lisa Cronkite, BS; Julie Currin, MD; Maureen Daly, RN; Caitlin Egan, MS; Erica Ferguson, BS, RD; Linda Foss, MPH; Jennifer Gauvin, BS; Don Kieffer, PhD; Lauren Lessard, BS; Deborah Maier, MS; JP Massaro, BS; Tammy Monk, MS; Rob Nicholson, PhD; Erin Patterson, BS; Suzanne Phelan, PhD; Hollie Raynor, PhD, RD; Douglas Raynor, PhD; Natalie Robinson, MS, RD; Deborah Robles; Jane Tavares, BS.

The University of Texas Health Science Center at San Antonio: Helen P. Hazuda, PhD^1^; Maria G. Montez, RN, MSHP, CDE^2^; Carlos Lorenzo, MD^3^; Charles F. Coleman, MS, RD; Domingo Granado, RN; Kathy Hathaway, MS, RD; Juan Carlos Isaac, RC, BSN; Nora Ramirez, RN, BSN.

VA Puget Sound Health Care System/University of Washington: Steven E. Kahn, MB, ChB^1^; Anne Kure, BS^2^; Edward J. Boyko, MD, MPH^3^; Edward Lipkin, MD, PhD^3^; Dace Trence, MD^3^; Subbulaxmi Trikudanathan, MD, MRCP, MMSc^3^; Elaine Tsai, MD^3^; Brenda Montgomery, RN, MS, CDE; Ivy Morgan‐Taggart; Jolanta Socha, BS; Lonnese Taylor, RN, BS; Alan Wesley, BA.

Southwestern American Indian Center, Phoenix, Arizona and Shiprock, New Mexico: William C. Knowler, MD, DrPH^1^; Paula Bolin, RN, MC^2^; Tina Killean, BS^2^; Maria Cassidy‐Begay, BSND, RND^2^; Katie Toledo, MS, LPC^2^; Cathy Manus, LPN^3^; Jonathan Krakoff, MD^3^; Jeffrey M. Curtis, MD, MPH^3^; Sara Michaels, MD^3^; Paul Bloomquist, MD^3^; Peter H. Bennett, MB, FRCP^3^; Bernadita Fallis, RN, RHIT, CCS; Diane F. Hollowbreast; Ruby Johnson; Maria Meacham, BSN, RN, CDE; Christina Morris, BA; Julie Nelson, RD; Carol Percy, RN, MS; Patricia Poorthunder; Sandra Sangster; Leigh A. Shovestull, RD, CDE; Miranda Smart; Janelia Smiley; Teddy Thomas, BS.

University of Southern California: Anne Peters, MD^1^; Siran Ghazarian, MD^2^; Elizabeth Beale, MD^3^; Kati Konersman, RD, CDE; Brenda Quintero‐Varela; Edgar Ramirez; Gabriela Rios, RD; Gabriela Rodriguez, MA; Valerie Ruelas MSW, LCSW; Sara Serafin‐Dokhan; Martha Walker, RD.

### Coordinating center

Wake Forest University: Mark A. Espeland, PhD^1^; Judy L. Bahnson, BA, CCRP^3^; Lynne E. Wagenknecht, DrPH^1^; David Reboussin, PhD^3^; W. Jack Rejeski, PhD^3^; Alain G. Bertoni, MD, MPH^3^; Wei Lang, PhD^3^; David Lefkowitz, MD^3*^ Patrick S. Reynolds, MD^3^; Denise Houston, PhD^3^; Mike E. Miller, PhD^3^; Laura D. Baker, PhD^3^; Nicholas Pajewski, PhD^3^; Stephen R. Rapp, PhD^3^; Stephen Kritchevsky, PhD^3^; Haiying Chen, PhD, MM^3^; Valerie Wilson, MD^3^; Delia S. West, PhD^3^; Ron Prineas, MD^3^; Tandaw Samdarshi, MD^3^; Amelia Hodges, BS, CCRP^2^; Karen Wall^2^; Carrie C. Williams, MA, CCRP^2^; Andrea Anderson, MS; Jerry M. Barnes, MA; Tara D. Beckner; Delilah R. Cook; Valery S. Effoe, MD, MS; Melanie Franks, BBA; Katie Garcia, MS; Sarah A. Gaussoin, MS; Candace Goode; Michelle Gordon, MS; Lea Harvin, BS; Mary A. Hontz, BA; Don G. Hire, BS; Patricia Hogan, MS; Mark King, BS; Kathy Lane, BS; Rebecca H. Neiberg, MS; Julia T. Rushing, MS; Debbie Steinberg, BS; Jennifer Walker, MS; Michael P. Walkup, MS.

### Central resources centers

Central Laboratory, Northwest Lipid Metabolism and Diabetes Research Laboratories: Santica M. Marcovina, PhD, ScD^1^; Jessica Hurting^2^; John J. Albers, PhD^3^, Vinod Gaur, PhD^4^.

ECG Reading Center, EPICARE, Wake Forest University School of Medicine:

Elsayed Z. Soliman MD, MSc, MS^1^; Charles Campbell ^2^; Zhu‐Ming Zhang, MD^3^; Mary Barr; Susan Hensley; Julie Hu; Lisa Keasler; Yabing Li, MD.

Hall‐Foushee Communications, Inc.: Richard Foushee, PhD; Nancy J. Hall, MA.

Federal Sponsors

National Institute of Diabetes and Digestive and Kidney Diseases: Mary Evans, PhD; Van S. Hubbard, MD, PhD; Susan Z. Yanovski, MD.

National Heart, Lung, and Blood Institute: Lawton S. Cooper, MD, MPH; Peter Kaufman, PhD, FABMR; Mario Stylianou, PhD.

Centers for Disease Control and Prevention: Edward W. Gregg, PhD; Ping Zhang, PhD.

^1^Principal Investigator.

^2^Program Coordinator.

^3^Co‐Investigator.

All other Look AHEAD staff members are listed alphabetically by site.

Authors’ roles: Study design: KMB, RHN, KCJ, CHD, RC, AVS, CJC, CEL, XP, and SBK. Study conduct and data collection: KJ and AVS. Data analysis: RHN. Data interpretation: KMB, RHN, KCJ, CHD, RC, AVS, CJC, CEL, XP, and SBK. Drafting manuscript: KMB, RHN. Revising manuscript content: KMB, RHN, KCJ, CHD, RC, AVS, CJC, CEL, XP, and SBK. Approving final version of manuscript: All authors approved the final version. KMB and RHN take responsibility for the integrity of the data analysis.
